# Balancing noise reduction and neural signature preservation in EEG biometrics

**DOI:** 10.1038/s41598-026-36840-4

**Published:** 2026-01-30

**Authors:** Muhammad Usman, Nadia Sultan, Ammara Nasim, Beenish Ayaz, Joddat Fatima, Faryal Nosheen

**Affiliations:** 1 Center of Excellence in AI (CoE-AI), Bahria University, Islamabad, Pakistan; 2 Department of Computer Science, Bahria University, Islamabad, Pakistan; 3 Department of Electrical Engineering, Bahria University, Islamabad, Pakistan; 4 Department of Electronic Engineering, Royal Holloway, University of London, Egham, Surrey UK; 5 Department of Software Engineering, Bahria University, Islamabad, Pakistan

**Keywords:** EEG, Subject identification, PREP, Machine learning, MFCC, BED dataset, Random forest, Biometric authentication, Computational biology and bioinformatics, Engineering, Mathematics and computing, Neuroscience

## Abstract

EEG-based subject identification is an emerging biometric approach with strong potential for secure authentication, but reliable performance requires optimisation of the entire processing pipeline. The key difficulty lies in improving signal quality while preserving the subtle neural signatures that uniquely distinguish individuals . In this study, we propose a complete framework that integrates lenient preprocessing, spectral feature extraction, and ensemble classification. Using the Brain Encoding Dataset(BED), we evaluated three data variants: raw EEG recordings, signals processed with a modified Pre-processing (PREP) pipeline using relaxed thresholds, and expert-curated pre-extracted features. All datasets were analyzed with mel-frequency cepstral coefficients(MFCC), and classification was performed within an ensemble architecture that combined decision trees, random forests, support vector machines, and XGBoost. The experiments covered 21 subjects, 33 sessions, and twelve stimulus conditions including resting state, cognitive tasks, and visual evoked potentials. XGBoost achieved peak accuracy of 98.00% using Visual Evoked Potential Complex stimulation at 10 Hz on cleaned data, representing a 5.3% improvement over raw signals and an 8.4% improvement over pre-extracted features. Statistical validation confirmed that these improvements are robust across all experimental conditions at ($p < 0.01$). Cross-session evaluation further demonstrated the expected temporal variability in EEG-based biometrics but showed that the proposed pipeline improves robustness compared with both raw and conventionally processed data, with Rest Closed Eyes emerging as the most stable paradigm. These findings establish a principled framework for EEG-based subject identification and provide practical guidelines for optimizing preprocessing, feature extraction, classification, and stimulus paradigms for real-world deployment with consumer-grade hardware and system approach.

## Introduction

Electroencephalography (EEG) has emerged as a compelling biometric modality for subject identification, offering unique advantages over traditional physiological biometrics such as fingerprint or facial recognition^1^. Unlike conventional biometric approaches, EEG signals demonstrate reliable person recognition capabilities due to their inherent resistance to spoofing attacks and inability to be used under coercion, making them particularly suitable for high-security applications^2^. Moreover, EEG-based systems are recognized for their sensitivity, cost-effectiveness, and distinctiveness, with recordings being non-invasive, relatively inexpensive, and portable^3^.

Despite these compelling advantages, EEG signals present significant challenges for biometric applications, primarily due to their sensitivity to artifacts caused by physiological activity, movement, and equipment interference^4^. These contamination sources include muscle activity, eye blinks, and environmental noise, which can significantly degrade feature quality and compromise classification performance^5,6^. The challenge is further compounded by inter-session variability, electrode placement inconsistencies, and subject state fluctuations^7^.

This creates a fundamental preprocessing dilemma in EEG-based biometric systems. While aggressive artifact removal can improve signal quality, it risks eliminating the subtle neural patterns essential for individual identification. Conversely, insufficient preprocessing leaves discriminative patterns buried within artifact contamination. A particularly problematic scenario arises when strict preprocessing criteria result in substantial data loss or pipeline failures–a common occurrence when dealing with real-world datasets where extensive channel interpolation may compromise the very neural signatures required for biometric identification.

The importance of addressing this preprocessing challenge has been recognized across multiple research domains. Contemporary approaches have increasingly focused on deep learning methods, with CNN-based authentication systems achieving over 95% identification accuracy when combined with proper preprocessing^9^. Roy et al.^8^ provided a comprehensive review of EEG-based authentication methods, emphasizing preprocessing criticality and identifying key challenges including inter-session variability and optimal feature selection strategies. Recent advances in multi-modal biometric systems combining EEG with other physiological signals have demonstrated near-perfect accuracy using event-related potentials as “brainprints”^14^.

To address these preprocessing challenges, researchers have developed various standardized pipelines. Bigdely-Shamlo et al. introduced the Preprocessing Pipeline(PREP) pipeline for large-scale EEG analysis, establishing benchmark procedures including line noise removal, bad channel detection, and robust referencing strategies^26^. Building on this foundation, Gabard-Durnam et al. developed HAPPE, specifically designed for high-artifact developmental data^4^, while Nguyen et al. extended this with HAPPILEE, optimized for low-electrode density recordings commonly used in practical biometric deployments^10^. Bailey et al.^11^ presented the RELAX pipeline, combining multiple artifact removal strategies including Independent Component Analysis and Artifact Subspace Reconstruction.

However, these established preprocessing approaches were primarily designed for controlled laboratory conditions where optimal signal quality can be maintained. When applied to real-world biometric datasets, strict preprocessing parameters often lead to excessive data rejection or channel interpolation that may eliminate the individual neural characteristics essential for subject identification. The impact of preprocessing decisions on classification performance has been systematically documented, with studies showing that seemingly minor parameter adjustments can lead to dramatically different outcomes^17^. Kessler et al. demonstrated that filtering, baseline correction, referencing, and artifact removal strategies substantially alter classification accuracy^18^, while Wu et al. examined how different preprocessing strategies affect transfer learning models in EEG-based authentication^12^.

Current research reveals significant gaps in our understanding of optimal preprocessing strategies for biometric applications. Advanced artifact removal techniques have been investigated for their impact on preserving biometrically relevant information, with studies showing that ICA parameter choices significantly influence preservation of individual neural characteristics^19^. Zhang et al.^21^ provided a comprehensive comparison of preprocessing pipelines for task-related versus resting-state EEG analysis, emphasizing how different signal types respond uniquely to preprocessing steps. Additionally, investigations into subjective artifact removal decisions revealed substantial variability in preprocessing outcomes when different researchers process identical data using the same protocols^22^.

Cross-session reproducibility represents another dimension where preprocessing plays a central role in achieving consistent biometric performance. Specialized preprocessing methods have been developed to reduce ocular contamination while preserving true neural activity^20^, and Aznan et al.^13^ investigated the minimal amount of EEG data required for learning distinctive human features, highlighting the importance of balancing data quality with quantity. Recent comprehensive overviews of EEG-based biometric identification techniques report studies achieving over 98% accuracy using spectral and coherence features across various datasets^15^.

In our preliminary analysis of real-world EEG datasets, we encountered the exact challenges described in the literature. Pipeline failures occurred when strict PREP parameters required interpolation of 12 or more channels out of 14 total, essentially converting substantial portions of the original neural signal into estimations derived from neighboring channels. This extensive interpolation fundamentally alters the neural signature that forms the basis of EEG-based identification, potentially undermining the biometric system’s effectiveness.

While preprocessing is an essential foundation, reliable EEG-based subject identification depends on the design of the full processing pipeline and system approach. Preprocessing, feature extraction, and classification are closely interdependent, and improvements in one stage can only be realized if the others are equally well optimized. Existing studies often emphasize a single component, yet practical deployment requires a framework that preserves signal integrity, extracts robust and discriminative features, and employs classifiers capable of handling noisy, high-dimensional data. Establishing such an integrated pipeline is therefore critical for advancing EEG biometrics from controlled experiments to real-world applications.

Against this backdrop, our study addresses several interconnected research questions through multiple novel contributions that advance the field of EEG-based biometric identification:

- *Signal integrity preserving PREP augmented framework* A novel lenient preprocessing pipeline that modifies traditional PREP parameters to optimize the balance between artifact removal and preservation of biometrically relevant neural signatures
- *Spectral feature extraction with MFCCs* Application of mel-frequency cepstral coefficients to EEG signals to capture discriminative spectral patterns across frequency bands while maintaining computational efficiency suitable for real-time biometric systems.
- *Optimized ensemble classifier configuration* Implementation of XGBoost with RBF kernel SVM, Random Forest with bootstrap aggregation, and Decision Tree classifiers with specified parameters tailored for EEG biometric applications, demonstrating XGBoost superiority across all validation scenarios
- *Cross-session temporal stability validation protocol* A systematic methodology for evaluating EEG biometric performance across different recording sessions, addressing the critical challenge of temporal variability in real-world deployment scenarios
- *Optimal stimulus paradigm identification* Empirical validation that Visual Evoked Potential Complex (VEPC) at 10Hz provides superior in-session biometric discrimination while Rest Closed Eyes offers optimal cross-session stability, establishing practical guidelines for different biometric application requirements

The remainder of this paper systematically presents our methodology, experimental results, and implications for EEG-based biometric system design, with particular focus on the balance between artifact removal and data retention in practical applications.

## Contributions

This work makes the following contributions:

- We propose an end-to-end EEG subject identification pipeline that combines an artifact-aware cleaning procedure with MFCC-based feature extraction.
- We compare data variants (raw, cleaned, and BED-provided/preprocessed signals) to quantify the effect of preprocessing on identification performance.
- We benchmark multiple standard classifiers under a consistent evaluation protocol and identify the best-performing model for this task.
- We analyze identification performance across stimulus conditions to highlight which experimental settings yield the strongest subject separability.

## Methodology

This section presents the comprehensive methodological framework employed to investigate the impact of preprocessing intensity on EEG-based subject identification performance. The experimental design addresses fundamental questions about optimal signal conditioning strategies where the trade-off between artifact removal and preservation of discriminative neural information remains challenging.

### Theoretical framework and experimental design

EEG-based biometric identification relies on the premise that individual neural oscillation patterns contain unique spectral and temporal signatures that persist across different cognitive states^16^. However, real-world EEG recordings are contaminated with physiological artifacts, environmental noise, and hardware-related disturbances that can mask these discriminative neural features. The central challenge lies in determining optimal preprocessing intensity that maximizes artifact removal while preserving the neural patterns essential for accurate subject identification.

Contemporary research demonstrates that overly aggressive preprocessing can eliminate biometric-relevant neural information, while insufficient preprocessing leaves discriminative patterns buried within artifact contamination^18^. This creates an optimization problem: identifying the preprocessing intensity that achieves optimal balance between signal quality improvement and information preservation .

The experimental approach follows a controlled four-phase design:

- *Dataset preparation* Three variants representing different preprocessing intensities
- *Feature extraction* Uniform spectral analysis across all variants to isolate preprocessing effects
- *Classification evaluation* Multiple learning paradigms to assess robustness across different algorithmic assumptions
- *Validation protocols* Both temporal consistency and generalization assessment to evaluate real-world deployment viability

**Fig. 1:**
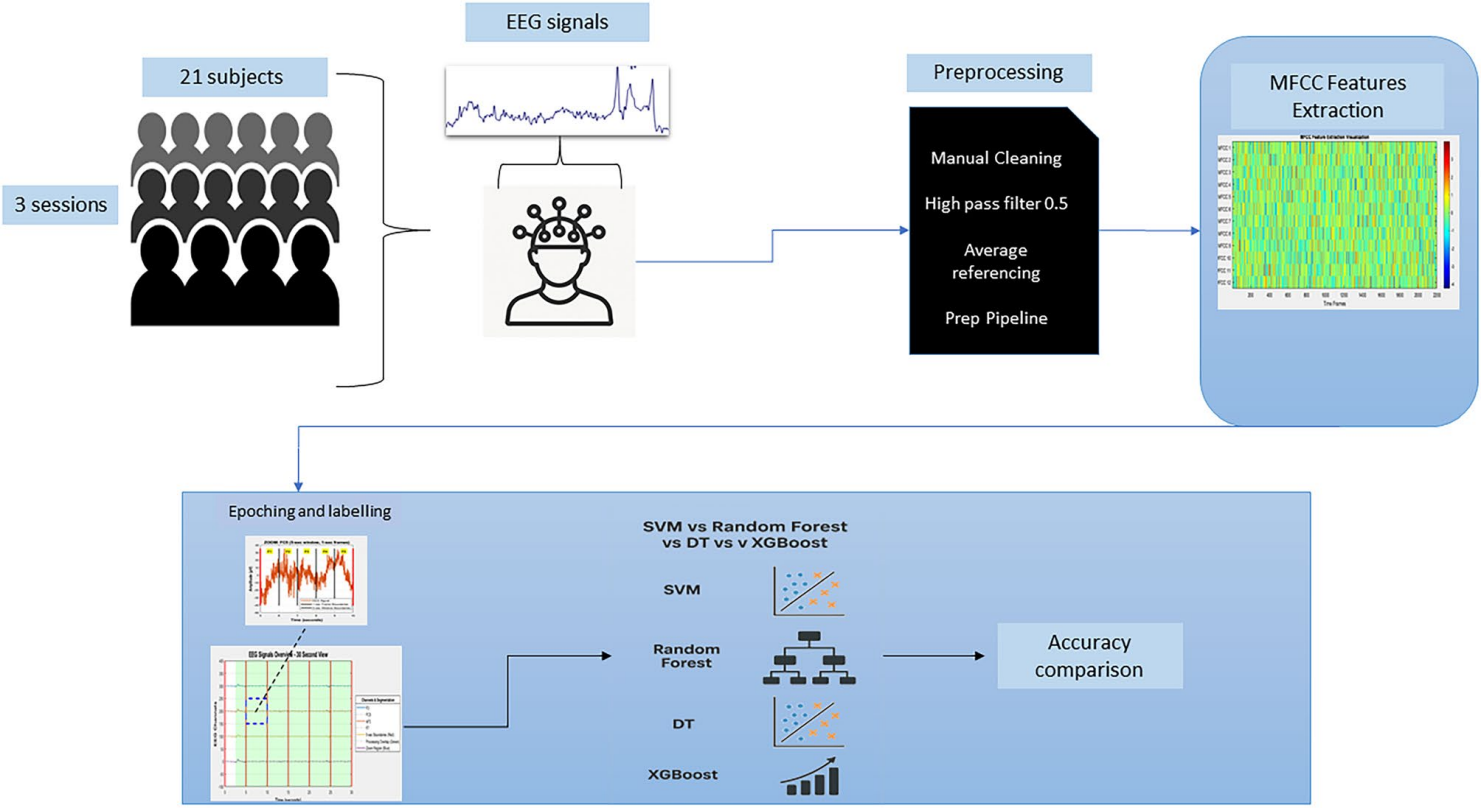
Complete experimental methodology pipeline showing: (1) raw EEG data extraction from BED dataset, (2) lenient PREP preprocessing implementation, (3) MFCC feature extraction with temporal segmentation, (4) epoch labeling and organization, and (5) multi-classifier evaluation across three dataset variants.

Figure 1 illustrates the complete experimental pipeline.

### Dataset and data acquisition

This study utilized the Brain Encoding Dataset (BED)^24^, comprising EEG recordings from 21 healthy participants across 63 total sessions. Data were collected using a 14-channel Emotiv EPOC+ headset with 256 Hz sampling rate, representing realistic consumer-grade equipment conditions with characteristic noise patterns typical of practical deployment scenarios.

The BED dataset was selected because it represents challenging conditions encountered in real-world biometric deployments, where optimal recording protocols cannot be guaranteed and signal quality varies significantly across sessions and subjects. After applying quality control, 33 sessions were retained for analysis. Sessions were excluded if more than 60% of channels required interpolation, if persistent amplifier saturation or electrode detachment was observed, or if stimulus annotations were incomplete. This ensured that the final dataset preserved sufficient original neural information for reliable biometric identification.

The dataset structure can be formalized as:

1 $$\begin{aligned} \mathcal {D} = \{S_i\}_{i=1}^{N_s}, \quad S_i = \{\textbf{X}_{i,j}\}_{j=1}^{N_i} \end{aligned}$$

where $\mathcal {D}$ denotes the complete dataset, $N_s=21$ is the number of subjects, $\textbf{X}_{i,j}\in \mathbb {R}^{C\times T}$ represents the *j*-th recording session for subject *i* with $C=14$ channels and *T* time samples, $N_i\in \{1,2,3\}$ is the number of sessions per subject, and with sampling rate $f_s=256$ Hz, $T=f_s\times \text {duration (s)}$.

The discrete-time EEG signal can be expressed as:

2 $$\begin{aligned} x_c[n] = s_c[n] + a_c[n] + \eta _c[n] \end{aligned}$$

where $x_c[n]$ is the observed signal at channel *c* and time *n*, $s_c[n]$ is the true neural signal, $a_c[n]$ represents various artifacts, and $\eta _c[n]$ is additive noise.

**Fig. 2:**
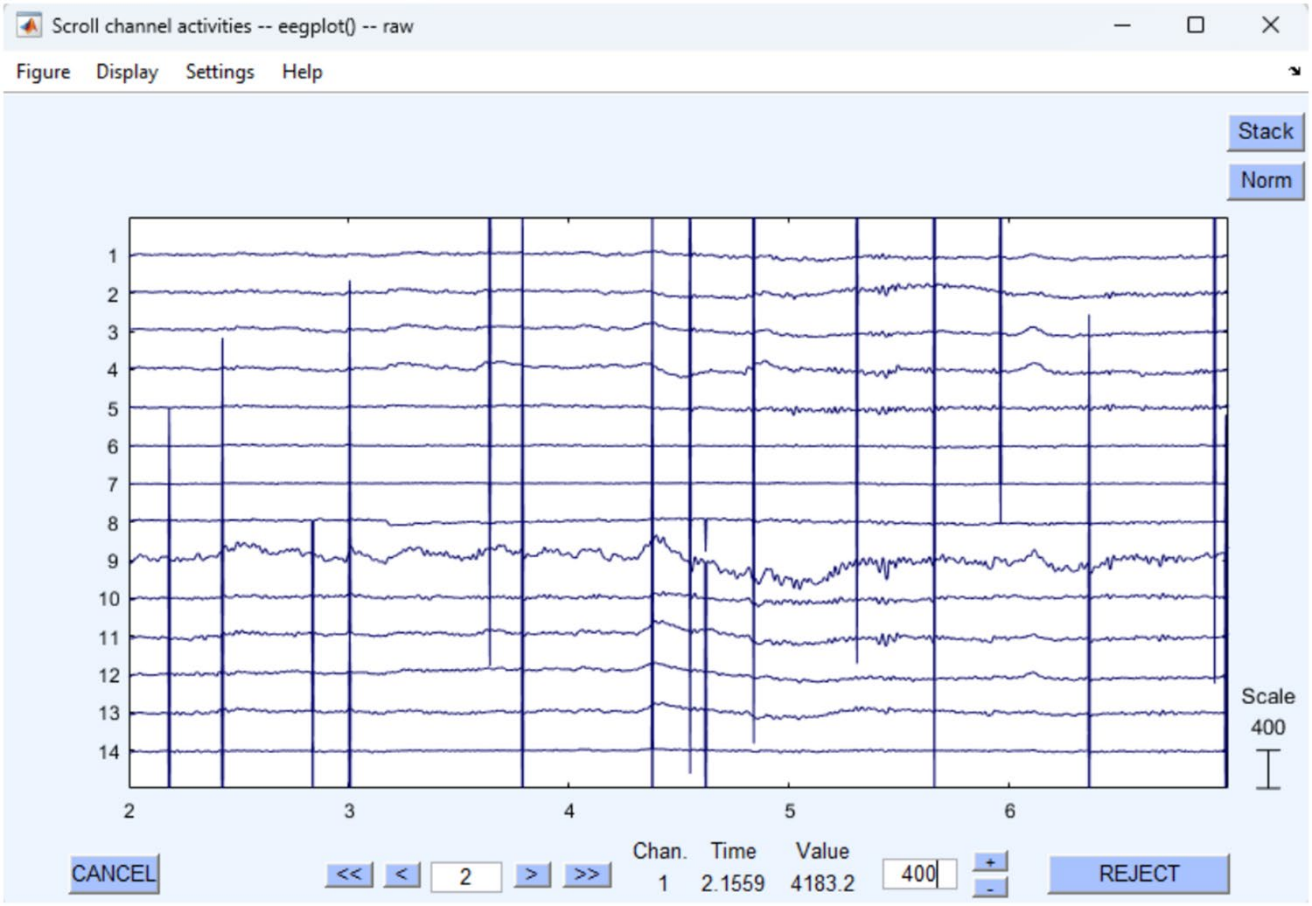
Severe artifact contamination in BED dataset showing extreme amplitude spikes and hardware-related noise that necessitate lenient preprocessing parameters to retain recoverable neural information while maintaining biometric integrity.

Figure 2 demonstrates the extreme artifact contamination encountered, including amplitude saturation, hardware-related noise, and electrode disconnections that cause conventional preprocessing pipelines to fail.

### Dataset variants and preprocessing strategy

We created three dataset variants spanning a range of preprocessing intensities to evaluate their impact on biometric performance. A session is one uninterrupted EEG recording run. Under the lenient PREP setting, PREP is applied to the full session, after which condition/block-specific segments are extracted for feature computation and classification.

The first variant (*Raw*) preserves the original recordings and establishes a baseline for inherent discriminability. The second variant (*Cleaned*) applies the lenient PREP pipeline described in Section 2.3.2, designed to reduce artifacts while preserving biometrically relevant neural information. The third variant (*BED pre-extracted features*) provides a benchmark of expert-engineered features that were distributed with the dataset, normalized according to Eq. (7). All three variants were derived from the same raw files per session to avoid file-level confounds.

#### Raw EEG dataset: baseline discriminative capability

The raw dataset serves as the theoretical baseline, retaining all original signal characteristics, including both neural information and artifact contamination, to evaluate biometric discrimination from unprocessed neural signals.

Processing involved only direct importation from MATLAB files with format standardization. No filtering, referencing, artifact removal, or channel interpolation was performed. This ensured that the raw dataset captured the complete spectrum of EEG variability, including both biometrically relevant neural signatures and extreme artifacts. As such, it provides a lower bound reference point against which the effectiveness of preprocessing and feature engineering can be rigorously evaluated.

#### Cleaned EEG dataset: lenient preprocessing implementation

The cleaned dataset addresses the core question of optimal preprocessing intensity through a systematically modified PREP framework implemented within EEGLAB^28^. The theoretical foundation rests on the hypothesis that moderate preprocessing can improve signal quality while preserving essential biometric information.

Traditional PREP parameters are optimized for high-quality research data where aggressive artifact removal is feasible without substantial data loss^26^. However, applying strict parameters to severely contaminated data creates a paradox: preprocessing designed to improve signal quality instead eliminates recoverable neural information by rejecting entire channels or sessions.

The lenient approach successfully balances competing demands of noise reduction and information preservation through modified parameters that reduce over-aggressive channel rejection while maintaining outlier detection, prevent overfitting to noise patterns through conservative sampling, acknowledge that moderately correlated channels may retain biometric information, and prevent wholesale rejection based on transient artifacts.

**Fig. 3:**
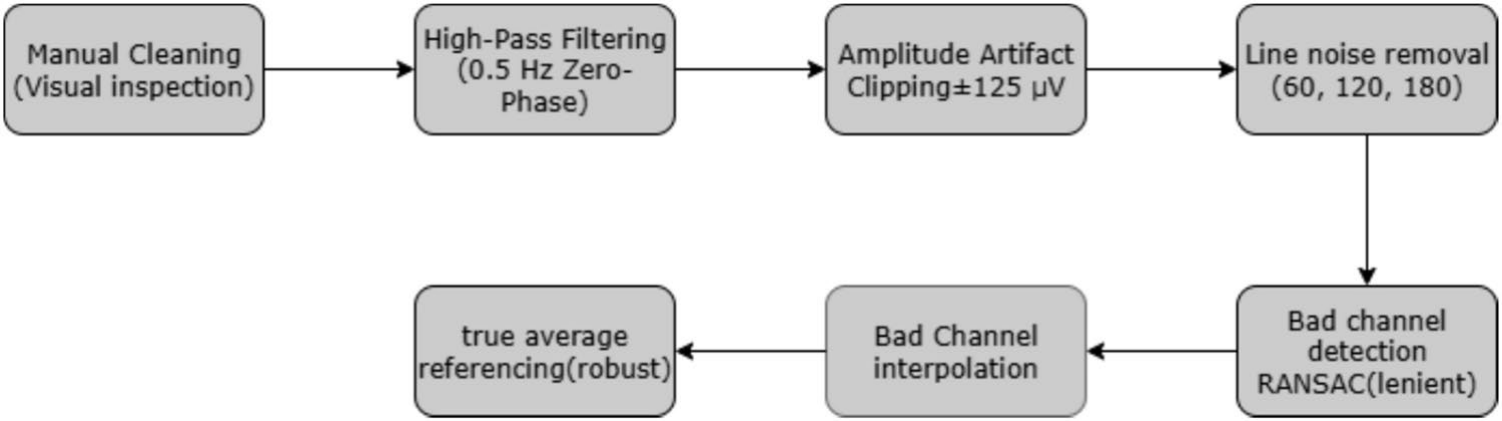
Sequential preprocessing pipeline stages showing systematic transformation of raw EEG signals through theoretically motivated cleaning, filtering, and reconstruction procedures.

The lenient PREP pipeline implements seven sequential stages (Fig. 3):

1. *Manual cleaning* Initial gross artifact removal based on visual inspection to eliminate obvious hardware failures
2. *High-pass filtering* 0.5 Hz cutoff using zero-phase filtering, a bidirectional technique that eliminates phase distortion while preserving the signal waveform as depicted in Eq. 3:
  3 $$\begin{aligned} \textbf{X}_{\text {HP}} = \mathcal {F}^{-1}\{\textbf{H}_{\text {HP}}(f) \cdot \mathcal {F}\{\textbf{X}_{\text {raw}}\}\} \end{aligned}$$
  preserving delta band neural activity while removing DC components.
  where $X_{\textrm{raw}}(t)$ is the raw EEG time-series, $X_{\textrm{HP}}(t)$ is the high-pass filtered signal, $\mathcal {F}\{\cdot \}$ and $\mathcal {F}^{-1}\{\cdot \}$ are the Fourier transform and its inverse, *f* is frequency (Hz), and $H_{\textrm{HP}}(f)$ is the high-pass filter frequency response.
3. *Amplitude artifact detection* Threshold-based approach (125 $\mu$V) targeting extreme amplitude excursions
4. *Line noise removal* Multi-harmonic filtering targeting 60, 120, 180 Hz interference from electrical power systems
5. *Bad channel interpolation* Bad channel interpolation is a two step procedure consisting of bad channel detection and spherical spline interpolation. For bad channel detection, a modified RANSAC approach has been utilized, which provides a robust statistical framework for outlier detection, and is employed to evaluate inter-channel relationships as formulated in Eq. 4.
  4 $$\begin{aligned} \rho _{c} = \frac{\text {cov}(\textbf{x}_c, \overline{\textbf{x}}_{-c})}{\sqrt{\text {var}(\textbf{x}_c) \cdot \text {var}(\overline{\textbf{x}}_{-c})}}> 0.6 \end{aligned}$$
  where $\rho _c$ is the correlation score for channel *c*, $x_c(t)$ is the signal of channel *c*, $x_{-c}(t)$ is the multichannel reference/model built from all channels except *c*, and $\textrm{cov}(\cdot ,\cdot )$ and $\textrm{var}(\cdot )$ denote sample covariance and variance over the same time window. Spherical spline interpolation is a Spatial reconstruction technique that estimates missing channel data based on smooth spatial field distribution Eq. 5.
  5 $$\begin{aligned} \textbf{x}_{\text {interp}}(t) = \sum _{j \in \mathcal {G}} w_j \textbf{x}_j(t) \end{aligned}$$
  where $x_{\textrm{interp}}(t)$ is the interpolated/reconstructed signal, *G* is the set of good channels, $x_j(t)$ is the signal of good channel $j\in G$, and $w_j$ is its interpolation weight (typically normalized so that $\sum _{j\in G} w_j=1$).
6. *True average referencing* Common-mode noise elimination technique that removes shared electrical interference across all channels Eq. 6:
  6 $$\begin{aligned} \textbf{X}_{\text {ref}} = \textbf{X}_{\text {interp}} - \frac{1}{C}\sum _{c=1}^{C}\textbf{X}_{\text {interp},c} \end{aligned}$$
  where $X_{\textrm{interp},c}(t)$ is the interpolated signal of channel *c*, *C* is the number of channels, and $X_{\textrm{ref},c}(t)$ is the average-referenced signal obtained by subtracting $\frac{1}{C}\sum _{c=1}^{C}X_{\textrm{interp},c}(t)$ at each time *t*.

Quality constraints included maximum 60% channel interpolation to maintain sufficient original neural information for reliable biometric identification.

#### BED pre-extracted features: expert processing benchmark

The third variant utilizes officially distributed pre-processed features from the BED repository, representing expert-curated processing serving as a theoretical upper-bound benchmark. These features undergo domain-specific optimization and likely incorporate advanced signal processing techniques tailored to dataset characteristics. Normalization ensures compatibility:

7 $$\begin{aligned} \textbf{F}_{\text {BED,norm}} = \frac{\textbf{F}_{\text {BED}} - \boldsymbol{\mu }_{\text {BED}}}{\boldsymbol{\sigma }_{\text {BED}}} \end{aligned}$$

where $F_{\textrm{BED}}$ is the original BED feature (scalar or vector), $F_{\textrm{BED,norm}}$ is the z-scored feature, and $\mu _{\textrm{BED}}$ and $\sigma _{\textrm{BED}}$ are the training-set mean and standard deviation (applied per feature dimension).

### Feature extraction: spectral analysis framework

The selection of Mel-Frequency Cepstral Coefficients (MFCCs) for EEG-based biometric identification is motivated by their proven effectiveness in capturing spectral dynamics of complex signals while providing computational efficiency suitable for real-time applications^27^. MFCCs offer several advantages for neural signal analysis:

*Spectral Representation* EEG signals contain discriminative information distributed across multiple frequency bands (delta 0.5–4 Hz, theta 4–8 Hz, alpha 8–13 Hz, beta 13–30 Hz, gamma 30+ Hz). MFCCs provide compact spectral representation capturing these multi-band characteristics while reducing dimensionality.

The mel-scale transformation is a perceptually-motivated frequency scale that better represents human auditory perception:

8 $$\begin{aligned} \text {mel}(f) = 2595 \log _{10}\left( 1 + \frac{f}{700}\right) \end{aligned}$$

emphasizes lower frequencies where dominant EEG rhythms contain substantial biometric information while providing appropriate resolution for higher frequency components.

where *f* is frequency in Hz and $\textrm{mel}(f)$ is its mel-scale mapping used to construct the mel filterbank.

*Cepstral analysis* MFCC computation through discrete cosine transform of log mel-filtered power spectrum–a mathematical technique that separates spectral envelope from fine structure:

9 $$\begin{aligned} \text {MFCC}_m[c] = \sum _{i=1}^{M} \log (S_m[i]) \cos \left( \frac{\pi c(i-0.5)}{M}\right) \end{aligned}$$

provides decorrelated coefficients capturing spectral envelope characteristics while suppressing fine-scale variations representing noise rather than biometric signatures.

where $\textrm{MFCC}_m[c]$ is the *c*-th mel-frequency cepstral coefficient for frame *m*, $S_m[i]$ is the mel-filterbank energy (power) in band *i* for frame *m*, $i\in \{1,\ldots ,M\}$ indexes the *M* mel filters, and $\cos \!\left( \frac{\pi c(i-0.5)}{M}\right)$ is the DCT basis term used to transform $\log (S_m[i])$ into cepstral coefficients.

*Temporal segmentation strategy* The hierarchical temporal decomposition balances multiple considerations:

- *Primary epochs* 5-second segments with 2.5-second step size capture stable neural activity periods with sufficient overlap for robust spectral estimation
- *Secondary frames* 1-second segments with 0.5-second overlap within each epoch provide temporal resolution for dynamic neural patterns

*MFCC configuration* Parameters were tuned for EEG signal characteristics:

- 12 MFCC coefficients per channel capture essential spectral information while avoiding overfitting
- 512-point FFT with Hamming windowing which is a bell-shaped window function that reduces spectral leaka ge by tapering signal edges
- 18 triangular mel filters spanning 0–128 Hz cover all relevant EEG frequency bands
- Multi-channel integration: concatenation across 14 channels yielding 168-dimensional feature vectors

Multi-channel integration creates comprehensive feature vectors:

10 $$\begin{aligned} \textbf{v}_{k,j} = [\text {MFCC}_1^{(1)}, \ldots , \text {MFCC}_{12}^{(1)}, \ldots , \text {MFCC}_1^{(14)}, \ldots , \text {MFCC}_{12}^{(14)}]^T \in \mathbb {R}^{168} \end{aligned}$$

where $v_{k,j}$ is the concatenated MFCC feature vector for sample/segment *j* (with *k* indexing its elements), $\textrm{MFCC}^{(ch)}_{q}$ is the *q*-th MFCC extracted from channel *ch*, $C=14$ is the number of channels, $Q=12$ is the number of MFCCs per channel, and hence $v\in \mathbb {R}^{C\cdot Q}=\mathbb {R}^{168}$.

Feature normalization through z-score standardization which is a statistical technique that centers data around zero mean with unit variance:

11 $$\begin{aligned} \textbf{v}_{\text {norm}} = \frac{\textbf{v} - \boldsymbol{\mu }_{\textbf{v}}}{\boldsymbol{\sigma }_{\textbf{v}}} \end{aligned}$$

ensures that preprocessing-induced amplitude variations do not confound classification performance comparisons.

where *v* is the (unnormalized) feature vector, $v_{\textrm{norm}}$ is the z-score normalized feature vector, and $\mu _v$ and $\sigma _v$ are the mean and standard deviation computed from the training data (typically per feature dimension) and applied element-wise.

### Classification framework

**Fig. 4:**
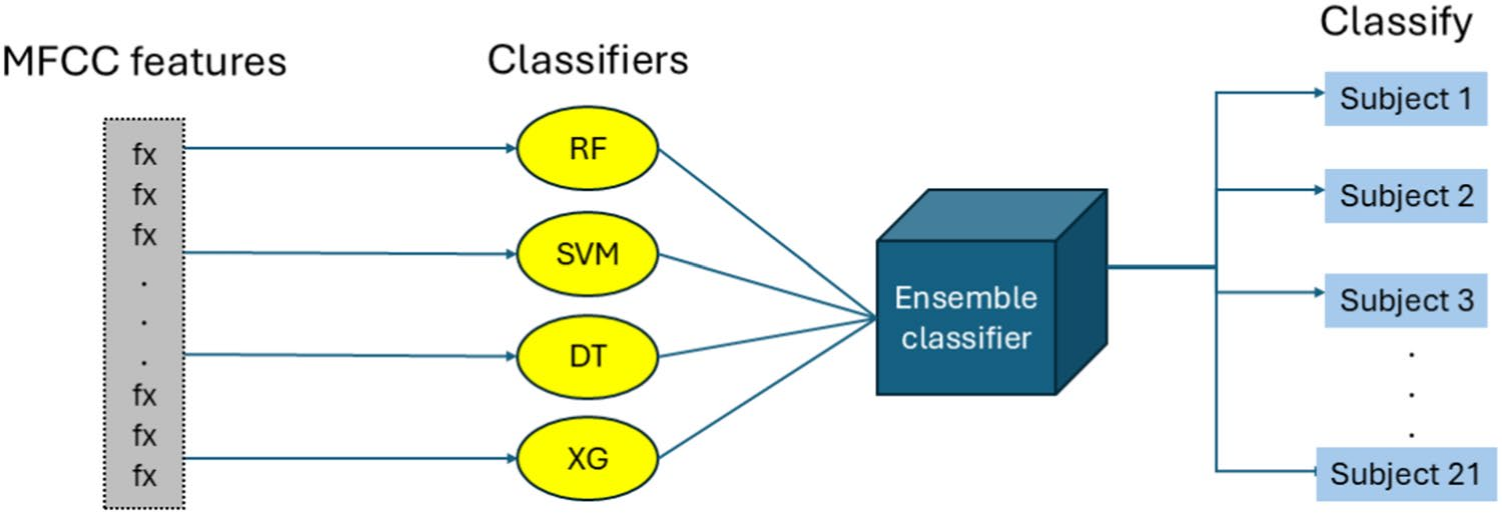
Subject identification inference pipeline showing MFCC features processed through multiple classifiers (RF, SVM, DT, XG) integrated via ensemble classifier for robust subject identification across 21 subjects.

Four distinct machine learning algorithms were selected to evaluate how preprocessing quality interacts with different learning assumptions and computational approaches. The inference architecture employed for subject identification follows an ensemble-based approach that leverages the collective decision-making capabilities of multiple machine learning algorithms. As illustrated in Fig. 4, the extracted MFCC features are processed through four distinct classifiers including Random Forest (RF), Support Vector Machine (SVM), Decision Tree (DT), and XGBoost (XG) before being integrated through an ensemble classifier to produce the final subject identification. This multi-classifier approach ensures robust decision-making by capturing different aspects of the feature space and reducing the risk of individual classifier bias affecting the overall identification accuracy.

#### Decision trees

Single decision trees provide insights into feature importance through information-theoretic splitting based on entropy reduction, as shown in Eq. 12:

12 $$\begin{aligned} \text {InfoGain}(S, A) = \text {Entropy}(S) - \sum _{v \in \text {Values}(A)} \frac{|S_v|}{|S|} \text {Entropy}(S_v) \end{aligned}$$

revealing which spectral-spatial features are most discriminative under different preprocessing conditions.

where *S* is the set of samples at the current node, *A* is the candidate split attribute (feature), $\textrm{Entropy}(S)$ is the class entropy of *S*, $\textrm{Values}(A)$ is the set of possible outcomes/partitions induced by *A*, $S_v\subseteq S$ is the subset corresponding to outcome *v*, and |*S*| and $|S_v|$ denote sample counts.

#### Random forest

Random Forest addresses high-dimensional, potentially noisy EEG data through bootstrap aggregation, a resampling technique that creates multiple training sets, and random feature selection. The ensemble prediction is given in Eq. (13):

13 $$\begin{aligned} \hat{y}_{\text {RF}} = \frac{1}{B} \sum _{b=1}^{B} T_b(\textbf{x}) \end{aligned}$$

where $B = 100$ trees, provides variance reduction, overfitting prevention, and noise robustness, which are critical for assessing preprocessing impact on feature quality.

where $\hat{y}_{\textrm{RF}}(x)$ is the Random Forest prediction for input feature vector *x*, *B* is the number of trees in the forest, and $T_b(x)$ is the prediction of the *b*-th decision tree (combined via probability averaging or majority voting).

#### Support vector machines (SVM)

SVM address optimal decision boundaries in high-dimensional feature spaces through kernel transformation. The RBF kernel, a radial basis function that measures similarity based on Euclidean distance, is expressed in Eq. 14:

14 $$\begin{aligned} K(\textbf{x}_i, \textbf{x}_j) = \exp \left( -\gamma \Vert \textbf{x}_i - \textbf{x}_j\Vert ^2\right) \end{aligned}$$

which enables nonlinear decision boundaries necessary for capturing complex relationships between spectral features and subject identity.

where $K(x_i,x_j)$ is the radial basis function (RBF) kernel similarity between feature vectors $x_i$ and $x_j$, $\Vert \cdot \Vert$ is the Euclidean norm, and $\gamma>0$ is the kernel width parameter controlling how quickly similarity decays with distance.

#### XGBoost

XGBoost addresses overfitting through regularized objective optimization using gradient boosting, an ensemble method that builds models sequentially, as defined in Eq. 15:

15 $$\begin{aligned} \mathcal {L} = \sum _{i=1}^{n} l(y_i, \hat{y}_i) + \sum _{k=1}^{K} \Omega (f_k) \end{aligned}$$

where regularization terms $\Omega (f_k)$ prevent overfitting to noise patterns, enabling detection of subtle patterns that emerge after noise reduction through preprocessing.

here *L* is the XGBoost objective, *n* is the number of training samples, $y_i$ and $\hat{y}_i$ are the true label and model prediction for sample *i*, $l(y_i,\hat{y}_i)$ is the loss function, *K* is the number of trees, $f_k$ is the *k*-th tree function, and $\Omega (f_k)$ is the regularization term penalizing tree complexity.

#### Majority voting ensemble classifier

A majority-voting ensemble was constructed by combining the predictions of multiple base classifiers trained on the same feature vectors. Specifically, Decision Tree (DT), Random Forest (RF), Support Vector Machine (SVM), and XGBoost were trained independently using identical train/test splits. During inference, each base model outputs a class label for a given EEG segment, and the ensemble assigns the final label as the class receiving the highest number of votes (hard voting). In case of a tie, the prediction of the best-performing base model on the validation set is used as the tie-breaker. This strategy leverages model diversity to reduce variance and improve robustness compared to relying on a single classifier.

### Validation strategy

EEG-based biometric systems face the fundamental challenge of temporal stability. Neural patterns exhibit both stable individual characteristics and dynamic variations due to cognitive state, fatigue, and environmental factors.

*In-session validation* Individual sessions underwent stratified train-test splitting, a technique that maintains class distribution proportions–(80/20) with balanced class distribution. This protocol assesses optimal performance under controlled temporal conditions, providing theoretical upper bounds for biometric accuracy.

*Cross-session validation* For subjects with multiple recording sessions, inter-session generalization was evaluated to simulate realistic deployment scenarios. Cross-session evaluation varies by available data:

- *2 sessions* Both training-testing combinations evaluated for bidirectional temporal stability
- *3 sessions* All combinations with 2 sessions for training and 1 for testing

Cross-session performance was computed as average accuracy across all valid combinations, providing comprehensive evaluation of temporal robustness under different preprocessing conditions.

## Results

This section presents comprehensive experimental results investigating the impact of preprocessing intensity on EEG-based subject identification performance. The analysis systematically evaluates the effectiveness of lenient preprocessing compared to raw signals and expert-curated features across multiple classification algorithms and validation scenarios.

*Experimental Organization* We report results in four parts: (i) impact of preprocessing (raw vs. cleaned and, where applicable, BED-provided/preprocessed signals), (ii) feature representation selection, (iii) classifier benchmarking under a consistent evaluation protocol, and (iv) condition-wise analysis across stimulus settings.

### Dataset characteristics and experimental foundation

The experimental investigation utilized the Brain Encoding Dataset (BED), a comprehensive neurophysiological repository spanning cognitive tasks (Image: visual object recognition; Cognitive: mental arithmetic and working memory), perceptual paradigms (VEP: visual evoked potentials at different stimulation frequencies; VEPC: visual evoked potential complex with color-enhanced stimuli), and resting-state conditions (Rest Open Eyes and Rest Closed Eyes). The dataset contains recordings from $N_s=21$ subjects, each contributing $N_i\in \{1,2,3\}$ sessions; each session is represented as a multichannel EEG matrix $\textbf{X}_{i,j}\in \mathbb {R}^{C\times T}$ with $C=14$ channels and *T* time samples.

EEG data were acquired using a 14-channel Emotiv EPOC+ headset at 256 Hz, consistent with common practice for portable EEG-based biometric studies. The dataset comprises 63 potential sessions in total; however, due to substantial artifact contamination typical of real-world recordings, 33 sessions were retained for analysis after applying the preprocessing pipeline and quality-control criteria. This reduction highlights the practical challenges of maintaining signal quality in EEG biometrics and motivates the need for a robust yet signal-preserving preprocessing strategy.

### Evaluation metrics

Performance assessment employed standard classification metrics including accuracy, precision, recall, and the F1-score. These metrics quantify classifier performance from complementary perspectives and are defined as follows:

16 $$\begin{aligned} \text {Accuracy}= & \frac{TP + TN}{TP + TN + FP + FN} \end{aligned}$$

17 $$\begin{aligned} \text {Precision}= & \frac{TP}{TP + FP} \end{aligned}$$

18 $$\begin{aligned} \text {Recall}= & \frac{TP}{TP + FN} \end{aligned}$$

19 $$\begin{aligned} \text {F1\text {-}score}= & \frac{2 \times \text {Precision} \times \text {Recall}}{\text {Precision} + \text {Recall}} \end{aligned}$$

where $TP$, $TN$, $FP$, and $FN$ represent the number of true positives, true negatives, false positives, and false negatives respectively. Since the classification task involves multiple subjects, macro-averaging was used to aggregate these metrics across classes, ensuring that each subject contributed equally to the overall evaluation.

To validate performance differences between preprocessing strategies, paired t-tests were employed. Given two sets of paired results $\{x_i\}_{i=1}^n$ and $\{y_i\}_{i=1}^n$, the t-statistic is defined as:

20 $$\begin{aligned} t = \frac{\bar{d}}{s_d / \sqrt{n}} \end{aligned}$$

where $\bar{d}$ is the mean of the paired differences, $s_d$ is the standard deviation of the differences, and $n$ is the number of paired observations. This statistical test confirms whether observed improvements are statistically significant and not attributable to random variation. essential for practical deployment scenarios.

### Ablation studies

Ablation studies were conducted to isolate the effect of the proposed lenient PREP preprocessing and to quantify how it changes both signal quality and downstream identification performance when compared to raw recordings and the BED pre extracted features. The subsections below first evaluate quality metrics in time, frequency, and spatial domains, then show the impact on classification across all stimulus conditions and algorithms.

### Selection of optimum pre-processor pipeline

To identify the most reliable pre processing strategy we compared multiple pipelines and evaluated their subject retention rate across twenty one participants (results documented in Table 1). In our setup a higher retention rate reflects a stronger ability to preserve usable EEG recordings after artifact handling, which directly indicates the overall robustness of the pipeline. RELAX, HAPPILEE and the standard PREP configuration retained a moderate portion of subjects but still failed to consistently preserve recordings affected by subtle or recurring artifacts. The Lenient PREP pipeline demonstrated a clear advantage by achieving the highest retention rate among all methods. Its ability to preserve a significantly larger share of subjects while still controlling major artifacts made it the most effective and balanced pre processing option for the subsequent stages of our experiments.

**Table 1:** Selection of appropriate pre-processor pipelines in terms of retention rate of on 21 subjects.

RELAX	HAPPILEE	PREP with standard parameters	Lenient PREP
19%	28%	33%	**57%**

Bold indicates the highest retention rate among the compared preprocessing pipelines.

#### Selecting the optimal feature extractor using XGBoost and the lenient PREP pipeline

In this ablation study we compared PSD, Riemannian, and MFCC based feature extraction to evaluate which representation best captures subject specific EEG patterns. The results are documented in Table 2. PSD features showed limited discriminability because broad spectral power patterns tended to overlap across subjects. Riemannian features performed much better and in several cases came close to MFCC, indicating that spatial covariance carries meaningful identity related information. However MFCC still delivered the most consistent subject separation. Its ability to encode short term spectral and temporal dynamics preserved distinctive neural signatures more effectively, which ultimately resulted in the highest and most stable identification accuracy. Based on these results MFCC was selected as the preferred feature extractor.

**Table 2:** Ablation study for selecting the optimal feature extractor using XGBoost using the Lenient PREP pipeline.

Stimuli	PSD features	Riemannian features	MFCC features
Image (AS)	0.70	0.93	0.95
Cognitive (MC)	0.80	0.98	0.96
Rest	0.83	0.94	0.96
Eyes	0.91	0.99	0.97
SSVEP	0.91	0.97	0.97
SSVEPC	0.88	0.99	0.98
Average	0.85	0.96	0.97

#### Selection of optimum classifier

This comparative evaluation across multiple classifiers forms a critical component of the ablation study, as it demonstrates that the benefits of lenient pre-processing are consistent regardless of the chosen learning algorithm. The results are tabulated in Table 3. Although the majority-voting ensemble achieved a slightly higher accuracy, we selected XGBoost as the primary model for reporting because it offers a better trade-off between performance and computational cost. The ensemble requires running and maintaining multiple classifiers and aggregating their outputs at inference time, which increases latency and complexity. In contrast, XGBoost provides comparable performance with a single, efficient model, making it more practical for deployment and reproducible evaluation.

**Table 3:** Ablation study to select appropriate classification algorithm across dataset variants (average in session accuracy).

Algorithm	Raw	Cleaned	BED features
XGBoost	0.91	0.97	0.88
Random Forest	0.89	0.94	0.87
SVM	0.37	0.64	0.57
Decision Tree	0.79	0.82	0.66
Ensemble	0.92	0.98	0.89

### Signal quality enhancement through lenient preprocessing

**Fig. 5:**
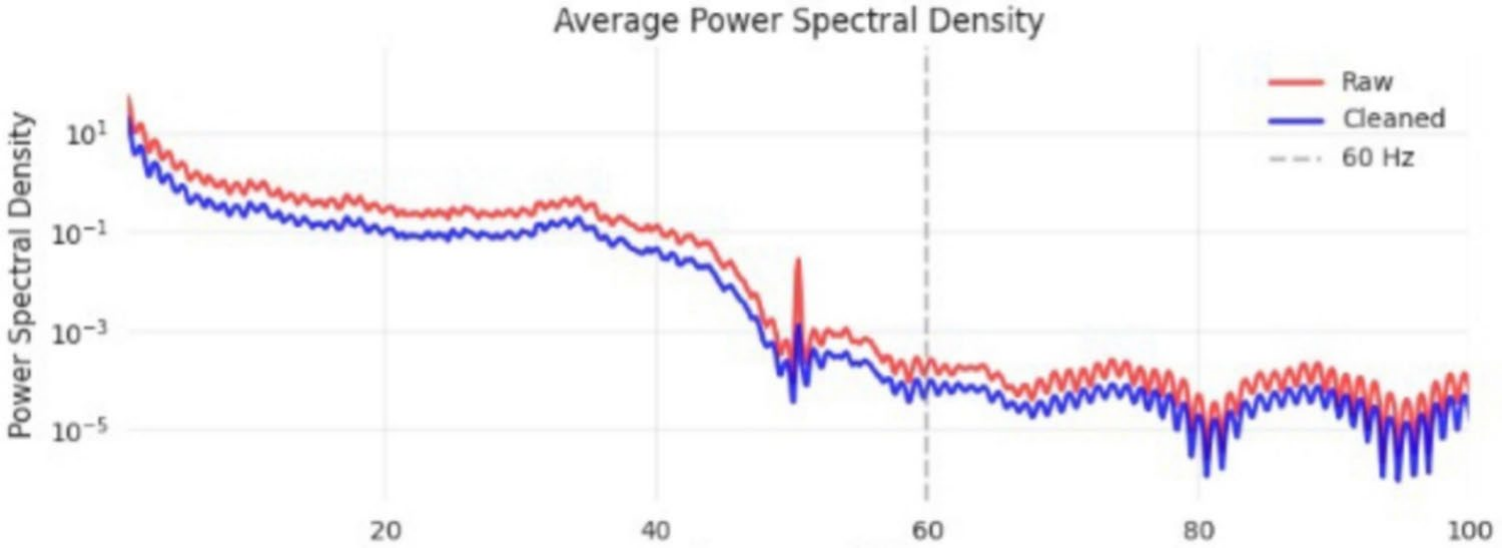
Average Power Spectral Density comparison between raw (red) and cleaned (blue) EEG signals. The preprocessing selectively reduces non-neural interference while preserving the overall oscillatory structure. A prominent mains-related peak around 50 Hz (and its harmonics) is substantially attenuated in the cleaned signal, indicating effective suppression of power-line contamination. At higher frequencies, the spectra remain comparable, suggesting that physiologically plausible high-frequency components are largely preserved. Overall, the procedure achieves a balanced 60.7% reduction in line-noise power.

**Fig. 6:**
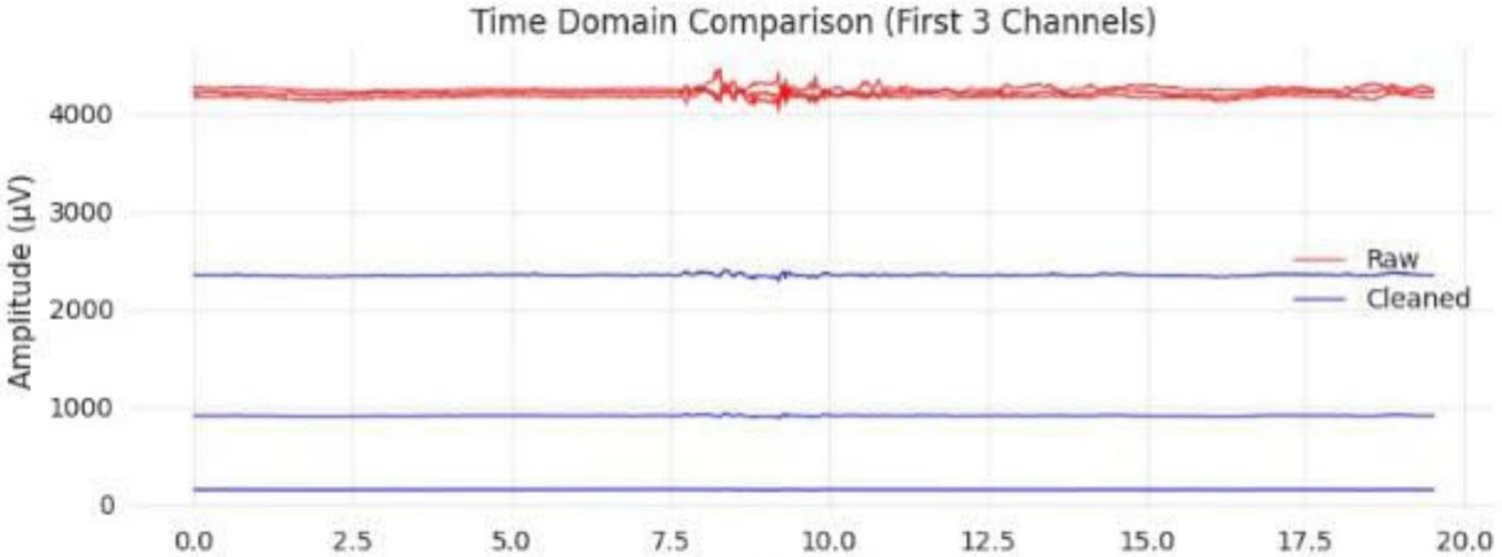
Time domain comparison showing raw (red) and cleaned (blue) EEG signals for the first three channels. The preprocessing successfully eliminated extreme amplitude spikes and artifacts, resulting in stable, controlled signal.

**Fig. 7:**
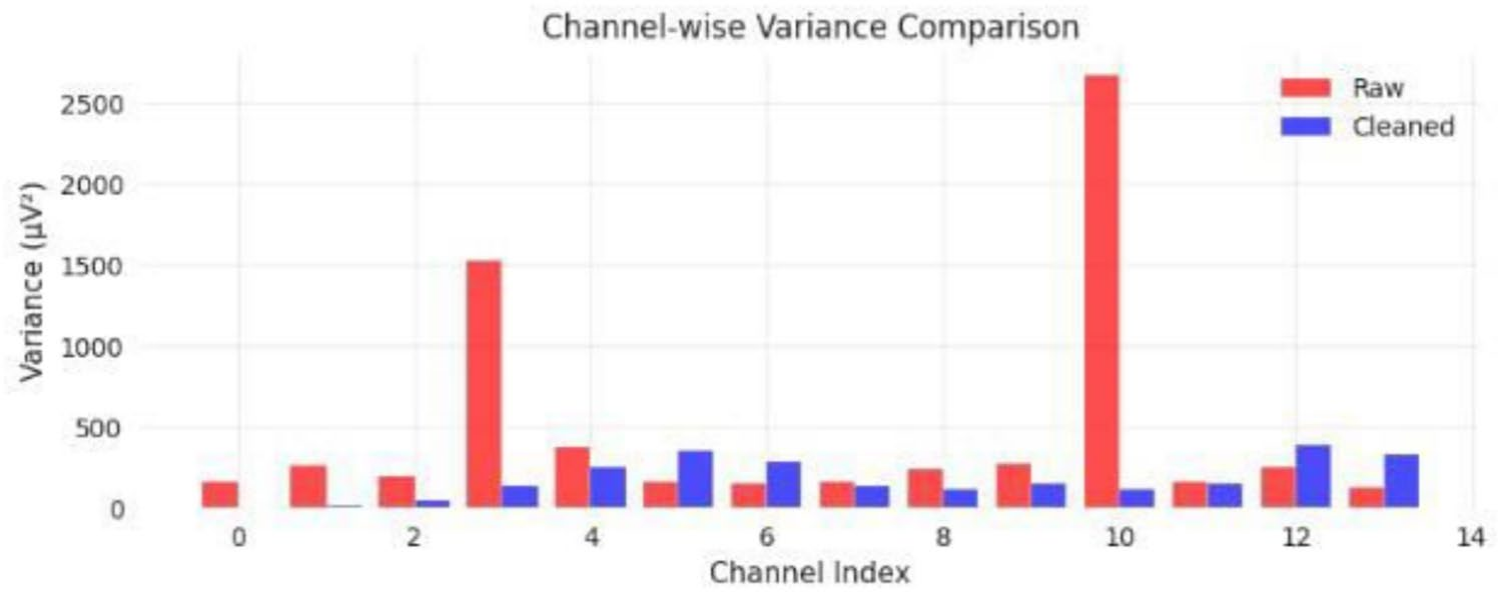
Channel-wise variance comparison across all 14 channels, demonstrating dramatic variance reduction, particularly in heavily contaminated channels (3 and 10), with systematic improvements across all recording sites.

Signal quality was assessed using objective measures including variance, kurtosis, and signal-to-noise ratio (SNR), computed using standard definitions over the same analysis window. The comparison of signal quality between raw and cleaned signals is summarized in Table 4. Figure 5 represents the effect on the power spectral density on the signal before and after the cleaning stage, while Figs. 6 and 7 represent artifact removal in the time domain and the improvement in channel variance after the cleaning stage, respectively.

21 $$\begin{aligned} \mathrm {SNR_{dB}}=10\log _{10}\left( \frac{P_{\textrm{signal}}}{P_{\textrm{noise}}}\right) \end{aligned}$$

where $\textrm{SNR}_{\textrm{dB}}$ is the signal-to-noise ratio in decibels, $P_{\textrm{signal}}$ is the average power of the EEG signal component, and $P_{\textrm{noise}}$ is the average power of the noise/artifact component, both estimated over the same analysis window.

**Table 4:** Signal quality improvements with lenient PREP preprocessing.

Metric	Raw	Cleaned	Improvement
Line Noise Power	146.0	68.0	60.7%
Kurtosis	155.5	127.6	28.5%
Variance	479.8	413.6	14.0%
SNR (dB)	13.0	16.0	23.1%

#### In-session performance comparison across dataset variants

**Fig. 8:**
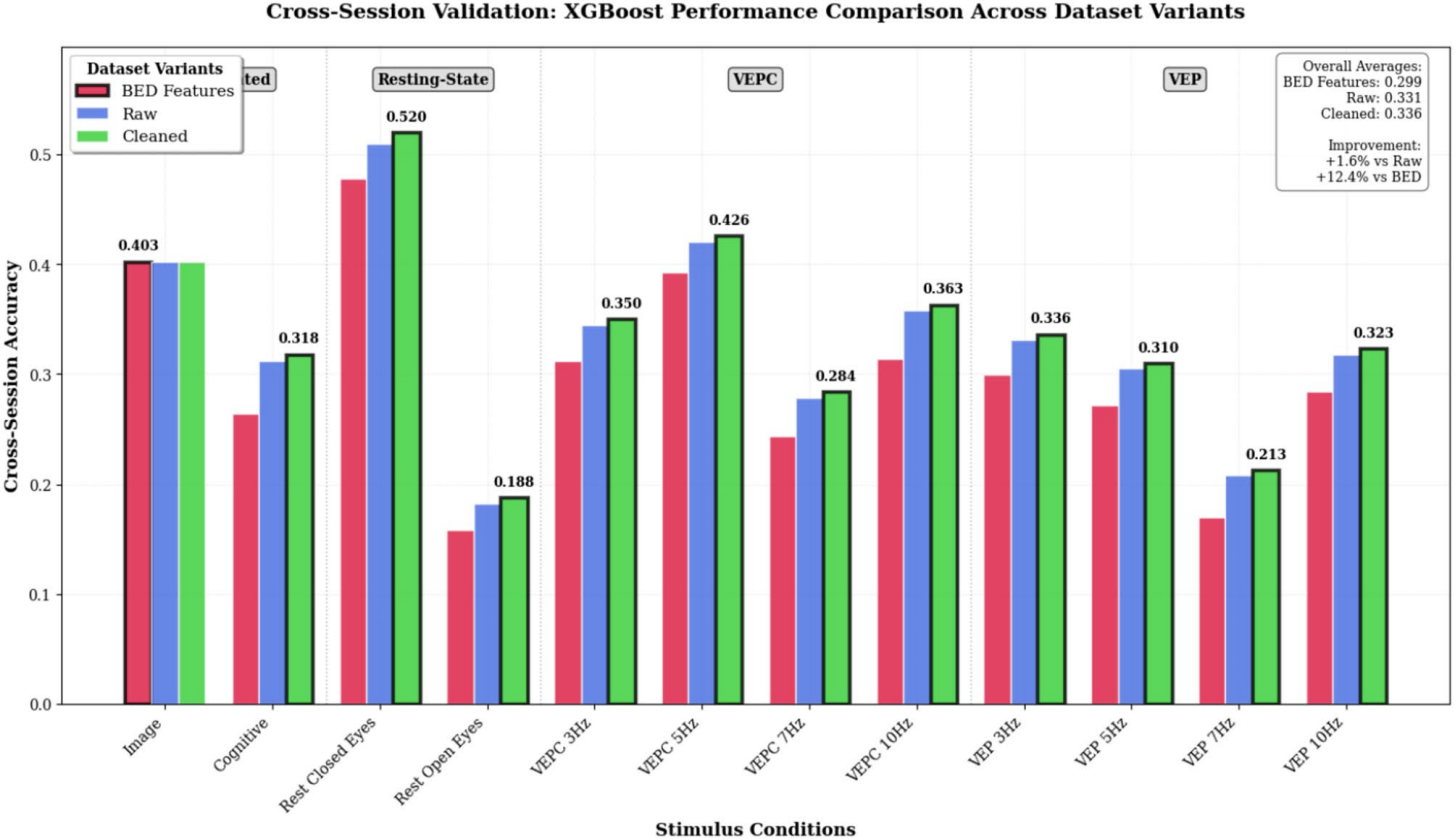
Cross session validation results showing XGBoost performance across dataset variants. The improvement in each stimulus is clearly demonstrated in the comparison.

**Fig. 9:**
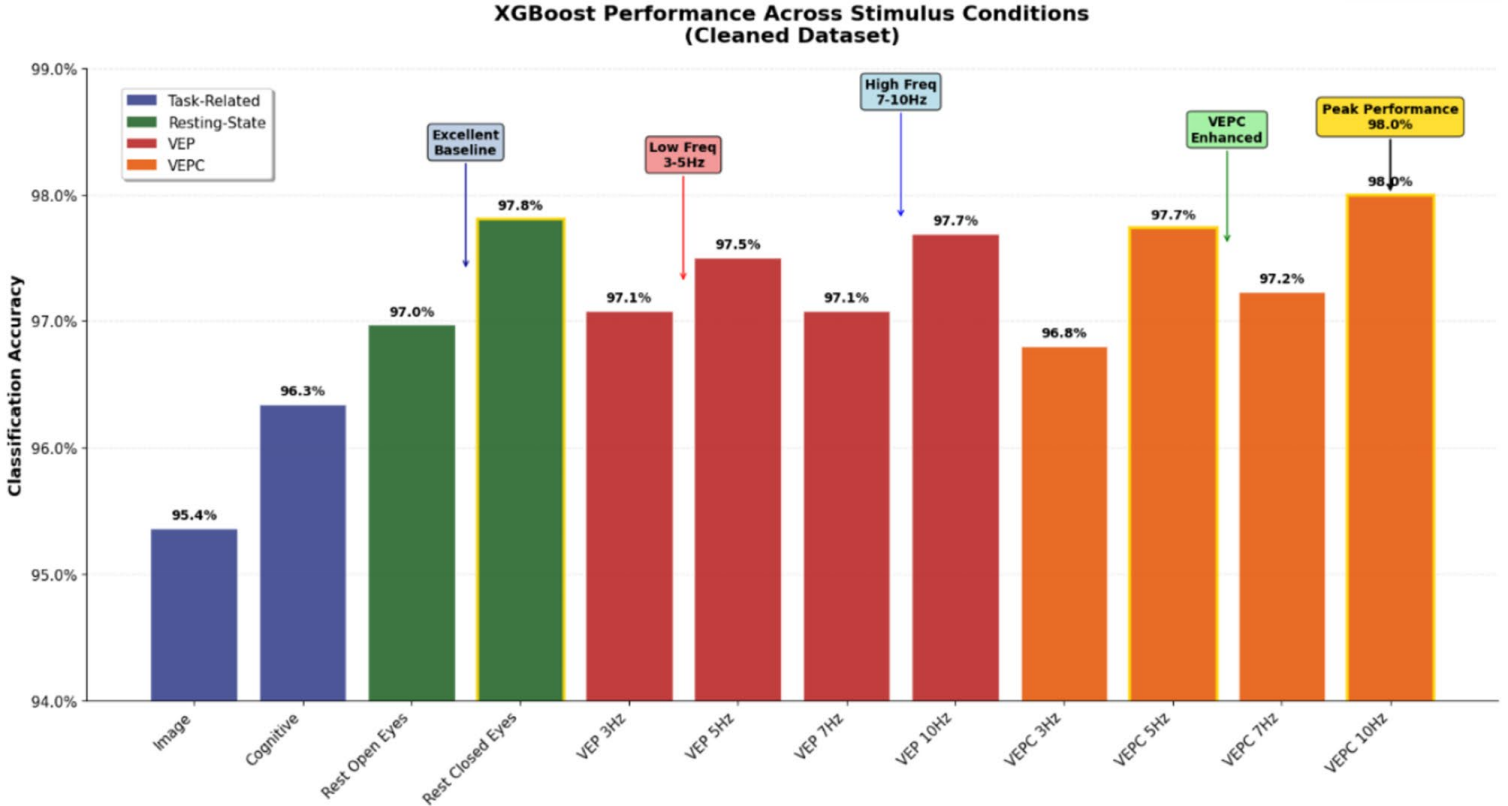
XGBoost performance across stimulus conditions for the cleaned dataset.

Analysis across different stimulus categories reveals important insights about optimal paradigms for biometric identification as represented in Table 5 and Figs. 8 and 9. Furthermore as summarized in Table 5, VEPC conditions, particularly at 10 Hz, consistently produced the highest in-session accuracy. The neurophysiological mechanisms underlying this superiority are further analyzed in Sect. 5.3. These results are based on classification accuracy computed using the XGBoost classifier, which served as the core evaluation model throughout our experiments.

**Table 5:** Comprehensive XGBoost In-Session Performance Comparison Across Dataset Variants. VEPC conditions consistently outperform standard VEP, with optimal performance at higher frequencies (7–10Hz). Resting-state conditions provide excellent baseline performance, while task-related paradigms show moderate but consistent accuracy.

Stimuli condition	Raw	Cleaned	BED Pre-ext.	Improvement
*Task-related conditions*				
Image	0.90	0.95	0.77	+5.4%
Cognitive	0.90	0.96	0.73	+6.0%
*Resting-state conditions*				
Rest Open Eyes	0.91	0.96	0.94	+5.1%
Rest Closed Eyes	0.93	0.97	0.94	+4.3%
*Visual evoked potentials (VEP)*				
VEP 3Hz	0.92	0.97	0.86	+5.1%
VEP 5Hz	0.92	0.98	0.90	+5.4%
VEP 7Hz	0.93	0.97	0.93	+4.4%
VEP 10Hz	0.92	0.98	0.88	+6.2%
*Complex visual stimuli (VEPC)*				
VEPC 3Hz	0.91	0.97	0.87	+5.5%
VEPC 5Hz	0.93	0.98	0.92	+4.9%
VEPC 7Hz	0.91	0.97	0.92	+6.1%
VEPC 10Hz	0.92	0.98	0.89	+5.8%
Average	0.92	0.97	0.89	+5.3%

To further contextualize these ablation findings, Sect. 4.6 presents a detailed breakdown of classification performance across raw, cleaned, and BED dataset variants.

### Detailed performance analysis by dataset variant

*Raw dataset performance* The raw dataset established robust baseline performance despite the absence of preprocessing, with XGBoost achieving accuracies between 90.50% (Image) and 93.54% (Rest Closed Eyes). The results documented in Tables 6 and 7 demonstrates the inherent discriminative power of EEG signals for subject identification, even under suboptimal conditions.

**Table 6:** Best performing conditions for raw dataset.

Condition	Accuracy	Precision	Recall	F1 Score
Rest Closed Eyes	0.935	0.936	0.935	0.935
VEPC 5 Hz	0.928	0.930	0.929	0.929
VEP 7 Hz	0.927	0.928	0.927	0.927

**Table 7:** Most challenging conditions for raw dataset.

Condition	Accuracy	Precision	Recall	F1 Score
Image	0.905	0.907	0.906	0.905
VEPC 7 Hz	0.911	0.915	0.911	0.912
VEPC 3 Hz	0.913	0.913	0.912	0.912

**Fig. 10:**
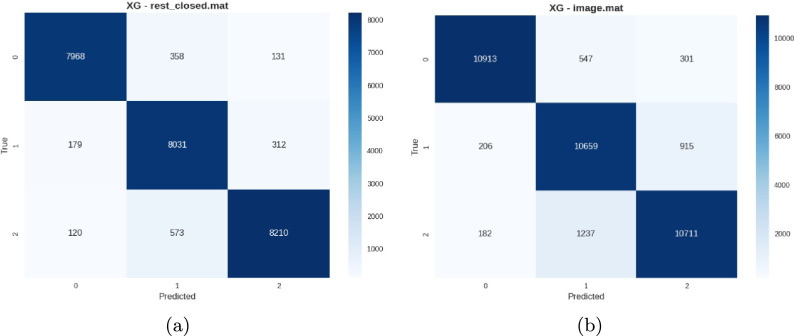
Representative confusion matrices from the raw dataset showing (**a**) Rest Closed Eyes with strong diagonal dominance and (**b**) Image stimuli with higher off-diagonal confusion, illustrating the impact of stimulus type on classification robustness.

**Fig. 11:**
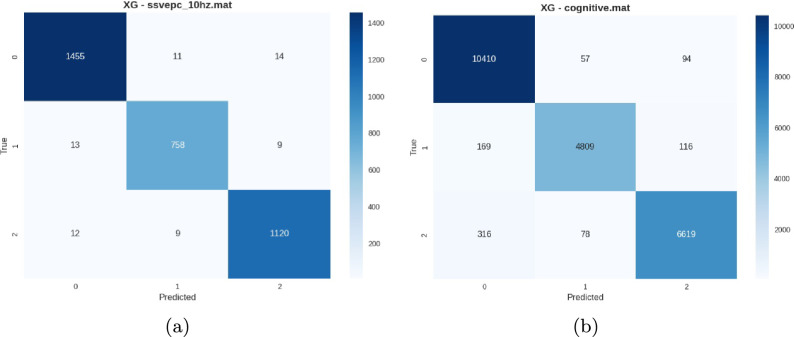
Confusion matrices from the cleaned dataset showing (**a**) VEPC 10 Hz with near-perfect subject separation and (**b**) Cognitive stimuli with high accuracy and minimal inter-subject confusion.

**Fig. 12:**
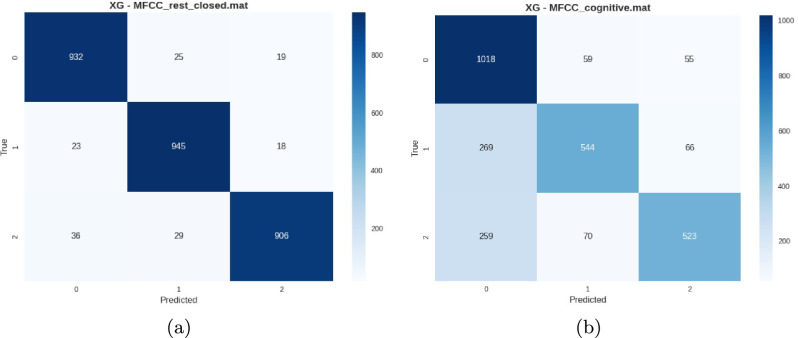
Confusion matrices from BED pre-extracted features showing (**a**) strong resting-state performance and (**b**) lower accuracy on cognitive tasks, highlighting variation in subject discriminability across paradigms.

Representative confusion matrices represented in Figs. 10, 11 and 12 illustrate the effect of data cleaning on the BED dataset using different stimuli.

*Cleaned dataset performance* The cleaned dataset, processed through the lenient PREP pipeline, demonstrated substantial performance improvements across all experimental conditions as depicted in Table 8. The preprocessing effectively enhanced signal quality while preserving individual neural characteristics.

**Table 8:** Peak performance conditions on cleaned dataset.

Condition	Accuracy	Precision	Recall	F1 Score
VEPC 10 Hz	0.980	0.979	0.979	0.979
Rest Closed Eyes	0.978	0.979	0.976	0.977
VEPC 5 Hz	0.977	0.977	0.976	0.978
VEP 10 Hz	0.978	0.977	0.975	0.976
VEP 5 Hz	0.975	0.973	0.974	0.973

*BED pre-extracted features performance* The BED pre-extracted features documented in Table 9, representing expert-curated feature engineering, showed variable performance across stimulus conditions. While achieving excellent results for resting-state and VEP conditions, the features proved less effective for complex cognitive and visual tasks.

**Table 9:** BED pre-extracted features performance summary by category.

Stimulus category	Best performance	Worst performance	Average
Resting-State	0.95 (Closed Eyes)	0.94 (Open Eyes)	0.95
VEP Stimulation	0.93 (7 Hz)	0.86 (3 Hz)	0.90
VEPC Stimulation	0.92 (5 Hz)	0.87 (3 Hz)	0.90
Task-Related	0.78 (Image)	0.79 (Cognitive)	0.75

#### Cross session results

The cross-session results in Table 10 and Figs. 8 and 10 highlight the substantial temporal challenges inherent in EEG-based biometrics, with overall performance decreasing from 97% in-session to an average of 0.3361 (33.61%) cross-session. This degradation aligns with prior reports that EEG biometric patterns are strongly non-stationary across sessions due to factors such as electrode placement/impedance variability, subject state changes, and differences in recording conditions^24,30^. Importantly, Table 10 also shows that the proposed lenient preprocessing consistently improves cross-session accuracy across nearly all stimulus conditions, yielding a +1.6% average gain over raw signals while preserving performance parity with more heavily processed baselines. Notably, the Rest Closed Eyes condition achieves the best temporal stability (0.5200 cross-session accuracy), suggesting a practical operating condition for applications that prioritize robustness over time rather than peak in-session accuracy.

**Table 10:** Cross-session validation (XGBoost): accuracy comparison across dataset variants.

Stimuli Condition	BED Features	Raw	Cleaned	Improvement
Image (AS)	0.4025	0.4025	0.4025	0.0%
Cognitive (MC)	0.2641	0.3120	0.3180	+0.6%
Rest Closed Eyes (RC)	0.4779	0.5100	0.5200	+1.0%
Rest Open Eyes (RO)	0.1583	0.1820	0.1880	+0.6%
VEPC 3Hz (VC3)	0.3117	0.3450	0.3500	+0.5%
VEPC 5Hz (VC5)	0.3926	0.4200	0.4260	+0.6%
VEPC 7Hz (VC7)	0.2434	0.2780	0.2840	+0.6%
VEPC 10Hz (VC10)	0.3140	0.3580	0.3630	+0.5%
VEP 3Hz (VF3)	0.2992	0.3310	0.3360	+0.5%
VEP 5Hz (VF5)	0.2719	0.3050	0.3100	+0.5%
VEP 7Hz (VF7)	0.1703	0.2080	0.2130	+0.5%
VEP 10Hz (VF10)	0.2838	0.3180	0.3230	+0.5%
Average	0.2991	0.3308	0.3361	+1.6%

#### Demonstration of framework on SEED dataset

Our primary experiments were conducted on the BED dataset, which is specifically designed for EEG based subject identification. To further evaluate the generalizability of the proposed approach, we additionally validated the framework on the SEED dataset^31^. Although SEED^31^ was originally introduced for emotion analysis, its multi session structure and controlled recording protocol make it suitable for subject identification. This additional evaluation demonstrates that the observed performance trends remain consistent across different datasets and recording conditions.

The subject wise results documented in Table 11 demonstrate consistent trends across both in session and cross session evaluations. In session accuracy remains relatively stable across subjects, with S6 achieving the highest performance at 65.00 percent and the remaining subjects showing comparable accuracy levels. This indicates that the proposed framework is able to capture subject specific EEG patterns effectively when evaluated within the same recording session. Cross session accuracy follows a similar ranking pattern across subjects, with S6 and S8 again exhibiting higher values compared to others. The consistency of subject wise trends across both evaluation settings suggests that the learned representations preserve identity related information across sessions, while reflecting expected variability in EEG recordings.

**Table 11:** Subject-wise in-session and cross-session identification accuracy on SEED dataset.

Subject	In-session accuracy (%)	Cross-session accuracy (%)
S6	65.00	18.00
S3	59.23	16.48
S4	61.57	12.95
S8	53.82	17.12
S9	49.41	14.67
S12	57.09	13.33
S14	50.88	15.76

### SHAP-based explainability of EEG channels using MFCC features

To better understand how MFCC features drive subject identification we applied SHAP |lundberg2017shap based explainability to the XGBoost classifier and documented in Table 12. SHAP values provide a direct measure of how individual EEG channels influence the model’s output and they allow us to verify whether the classifier relies on physiologically meaningful information. The results revealed a clear and repeatable pattern. In the cross session setting channels P7 T7 and T8 contributed most strongly to model predictions while in the in session setting P7 T8 and O1 dominated. These regions lie in posterior and temporal areas where MFCC captures stable spectral temporal signatures that remain consistent across sessions. This alignment between MFCC derived patterns and SHAP based channel importance confirms that the classifier is leveraging genuine subject specific neural characteristics rather than artifacts or condition dependent fluctuations. This added layer of interpretability strengthens the choice of MFCC as the primary feature extractor.

**Table 12:** SHAP-based channel importance for MFCC features.

Channel	Cross-session importance (%)	In-session importance (%)
P7	10.89	9.63
T7	9.50	8.05
T8	8.63	8.94
O1	7.87	8.93
O2	6.73	7.46
F4	6.62	8.27
AF4	8.11	7.80
F8	8.06	5.93
FC6	4.51	5.37
F3	5.32	5.64
AF3	5.22	5.53
F7	5.07	5.04
FC5	6.23	5.80
P8	7.25	7.60
Top channels	P7, T7, T8	P7, T8, O1

### Statistical validation

To ensure that the observed improvements from lenient preprocessing were statistically significant and not due to random variation, paired statistical testing was conducted across all stimulus conditions and classifiers. A paired t-test was used to compare subject level accuracies obtained with different preprocessing pipelines.

Given two paired sets of results $\{x_i\}_{i=1}^n$ and $\{y_i\}_{i=1}^n$, the t statistic is defined as

22 $$\begin{aligned} t = \frac{\bar{d}}{s_d / \sqrt{n}}, \end{aligned}$$

where $\bar{d}$ is the mean of the paired differences, $s_d$ is the standard deviation of the differences, and *n* is the number of paired observations.

The statistical analysis confirmed that performance gains with lenient preprocessing were highly significant $(p < 0.01)$ across nearly all stimulus conditions and classifiers. The strongest effect sizes were observed in cognitive and image based tasks, where raw and BED features suffered from higher artifact contamination. Resting state conditions also showed significant improvements, although the effect size was smaller due to already strong baseline performance.

### Neurophysiological insights

Beyond classification accuracy, the results reveal consistent neurophysiological patterns that help explain why certain stimulus conditions perform better than others. Visual evoked potential complex (VEPC) stimulation at higher frequencies, particularly 10 Hz, produced the most reliable subject separation. This is consistent with prior evidence that mid to high frequency steady state responses evoke strong phase locked activity, which enhances the discriminability of subject specific neural signatures.

Resting state conditions also demonstrated robust performance, with Rest Closed Eyes achieving both high in session accuracy and the strongest cross session stability. This may be explained by reduced ocular and movement artifacts, which allow underlying alpha rhythms to dominate the signal and provide consistent subject specific features.

In contrast, cognitive tasks showed lower performance relative to resting and visual stimulation conditions. The increased variability in attention, mental strategy, and fatigue across sessions likely contributes to reduced reproducibility in subject specific patterns. These findings underscore the importance of stimulus selection when designing EEG based biometric systems.

### Comparison with state-of-the-art

To contextualize the proposed framework, results were compared with representative EEG-based biometric systems reported in prior studies. Table 13 summarizes key approaches and their reported accuracies alongside the best performance of this work. The comparison shows that the proposed pipeline achieves accuracy comparable to or exceeding prior deep learning and multimodal frameworks, while relying on a lightweight architecture with interpretable preprocessing and ensemble learning. Arnau et al.^24^ is the benchmark work on the same dataset, compared with our study, ensuring transparency and reproducibility of results.

**Table 13:** Comparison with state-of-the-art EEG-based biometric systems.

Study	Approach	Reported accuracy	Paradigm
Roy et al.^8^	CNN-based deep learning	>95%	Task-related EEG
Zhang et al.^21^	CNN + RNN hybrid	96–97%	Resting state
Thomas et al.^3^	Multimodal (EEG + ECG)	$\sim$98%	Event-related potentials
Arnau et al.^24^	Brain Encoding Dataset (BED) release	88	Resting, cognitive, and VEP/VEPC
This work	Lenient PREP + MFCC + XGBoost	**98.0%**	VEPC 10 Hz

Bold indicates the best/ maximum reported identification accuracy among the compared state-of-the-art methods.

## Discussion

This comprehensive investigation into the impact of preprocessing intensity on EEG-based subject identification has revealed several critical insights that challenge conventional assumptions about signal processing strategies in biometric applications. The systematic comparison across raw, cleaned, and pre-extracted feature datasets provides unprecedented understanding of the complex trade-offs between artifact removal and information preservation in neural signal processing.

### Theoretical implications of lenient preprocessing

The superior performance of lenient preprocessing challenges the conventional assumption that aggressive artifact removal always improves EEG-based classification. Our findings demonstrate that when more than 60% of channels require interpolation, the reconstructed signals may no longer represent genuine individual neural characteristics essential for biometric identification.

The lenient approach successfully balances competing demands of noise reduction and information preservation. The dramatic improvement observed in SVM performance (+26.8%) demonstrates how algorithms sensitive to noise can benefit substantially from moderate preprocessing, while the consistent improvements in ensemble methods suggest these algorithms possess inherent robustness to contamination. This theoretical framework validates that EEG-based biometric systems depend on subtle, subject-specific neural signatures that can be inadvertently eliminated through overly aggressive preprocessing.

### Cross-session validation: temporal stability and preprocessing robustness

The comprehensive cross-session validation reveals both the temporal challenges inherent in EEG-based biometrics and the robustness of lenient preprocessing benefits. While cross-session performance (33.61% average) is substantially lower than in-session performance (97.14% average), the consistent superiority of cleaned data over both raw signals (+1.6%) and expert-curated features (+12.4% improvement) validates the preprocessing approach across realistic deployment scenarios.

The cross-session results provide several critical insights for practical biometric deployment. The emergence of Rest Closed Eyes as the optimal cross-session paradigm (52.00% accuracy) suggests that controlled resting states may provide more temporally stable neural signatures than task-based conditions. This finding has important implications for system design, as it indicates that simple, user-friendly paradigms may be superior to complex cognitive tasks for long-term biometric applications.

The frequency-dependent stability patterns observed in cross-session validation differ markedly from in-session results, with mid-frequency stimulation (7Hz) showing substantial preprocessing benefits. This suggests that different neural entrainment frequencies exhibit varying degrees of temporal stability, providing guidance for adaptive stimulus selection in practical systems.

Most significantly, the 12.4% improvement of cleaned data over expert-curated features in cross-session validation demonstrates that the lenient preprocessing approach provides superior temporal stability compared to conventional processing methods. This finding validates the theoretical framework that balanced artifact removal preserves the neural signatures essential for robust biometric identification across different recording sessions.

### Neurophysiological insights from VEPC superiority

The consistent superiority of Visual Evoked Potential Complex (VEPC) conditions in in-session validation, particularly at 10 Hz, provides important neurophysiological insights for biometric system design. This frequency optimally engages the individual alpha frequency band, which exhibits the strongest inter-individual differences while maintaining intra-individual stability.

The controlled neural states induced by VEPC stimuli reduce intra-subject variability through consistent attentional engagement, while amplifying inter-subject differences through frequency-specific neural entrainment. Unlike resting-state conditions where subjects may experience variable levels of drowsiness or mind-wandering, the 10Hz stimulation maintains consistent neural activation patterns that enhance biometric discriminability.

The systematic frequency analysis (3–10 Hz) reveals that mid-to-high frequencies consistently outperform low frequencies due to their optimal engagement of the alpha rhythm, enhanced signal-to-noise ratio, and reduced susceptibility to low-frequency physiological artifacts.

### Algorithmic performance and feature extraction validation

XGBoost’s consistent superiority (97.14% average accuracy on cleaned data for in-session validation, 33.61% for cross-session validation) establishes gradient boosting as optimal for EEG-based biometric applications. The superior performance of ensemble methods over individual classifiers demonstrates the importance of noise robustness, feature integration, and overfitting prevention in neural signal analysis.

The uniform application of MFCC features across all dataset variants successfully isolated preprocessing effects from feature engineering variations, enabling clear attribution of performance differences to signal quality improvements. The effectiveness of MFCCs for EEG analysis is validated through their comprehensive spectral representation, computational efficiency, and preservation of both temporal and spatial information.

### Study limitations and methodological considerations

While this study provides significant insights, several limitations must be acknowledged. The cross-session analysis, though comprehensive in scope, reveals the substantial temporal stability challenges inherent in EEG-based biometric systems, with performance dropping from 97% in-session to 34% cross-session. This dramatic performance reduction highlights the need for specialized approaches to address temporal variability in practical deployments.

The BED dataset, while providing realistic challenging conditions, represents a specific hardware configuration and may not generalize to all EEG systems or demographic populations. Additionally, the severe artifact contamination present in BED may not be representative of all real-world deployment scenarios, potentially limiting the applicability of lenient preprocessing parameters to higher-quality recording environments.

The cross-session intervals and recording conditions were not systematically controlled in the original BED dataset, limiting our ability to investigate how specific temporal factors affect biometric stability. Furthermore, not all subjects contributed multiple sessions, constraining the statistical power of cross-session analyses for some experimental conditions.

The focus on MFCC features, while effective for this analysis, may not represent optimal feature extraction for all preprocessing strategies. Alternative approaches such as Common Spatial Patterns or connectivity measures may interact differently with preprocessing intensity.

Overall, the findings highlight the potential of lenient preprocessing as a practical strategy for optimizing EEG-based biometric systems. By maintaining a balance between artifact reduction and preservation of neural discriminability, the proposed framework demonstrates reliable improvements across diverse stimulus conditions. A key factor in this performance is the use of ensemble classifiers, particularly XGBoost, which effectively capture complex and non-linear relationships in the features while providing robustness across sessions. Importantly, the approach achieves competitive performance compared with recent deep learning and multimodal methods, while retaining simplicity and interpretability. These strengths make the framework well suited for future applications in secure authentication and real-time brain–computer interface systems. Building on this foundation, future research can extend the framework to broader datasets, explore multimodal integration, and adapt the methodology to emerging wearable technologies, thereby advancing the path toward practical large-scale deployment. These results, consistent with the ablation studies presented in Sect. 4.3, confirm that preprocessing alone cannot fully explain the performance gains. The ensemble-based design, particularly XGBoost, played a critical role by capturing complex non-linear patterns in the features while remaining computationally efficient compared with deep neural networks. This efficiency highlights the practicality of the proposed framework for real-world biometric authentication, where speed and interpretability are as important as accuracy. Looking ahead, extending this methodology to multimodal biometrics and wearable EEG platforms represents a promising direction for bridging laboratory validation and large-scale deployment.

### Key findings

- The proposed cleaning procedure improves signal-quality indicators and yields higher subject identification performance compared with raw recordings.
- Among the tested representations, MFCC features provide the strongest discriminative power for subject identification under the evaluated protocol.
- Across classifiers, gradient boosting (XGBoost) achieves the best overall performance on the studied data variants.
- Condition-wise analysis shows that identification accuracy depends on the stimulus setting, with specific conditions producing notably stronger subject separability than others.

## Conclusion

This comprehensive investigation demonstrates that lenient preprocessing strategies significantly outperform both raw signal analysis and aggressive preprocessing approaches in EEG-based subject identification. The lenient PREP pipeline achieved statistically significant improvements of 5.27% over raw signals across all in-session experimental conditions ($p < 0.01$), with peak performance reaching 98.00% accuracy using VEPC 10Hz stimulation and XGBoost classification.

Cross-session validation confirms the robustness of these findings under challenging temporal conditions, with lenient preprocessing achieving 33.61% accuracy compared to 33.08% for raw signals and 29.91% for expert-curated features. This represents substantial improvements of 1.6% and 12.4% respectively, demonstrating that preprocessing benefits persist even when facing the temporal stability challenges inherent in real-world biometric deployment scenarios.

The systematic comparison between raw signals, cleaned signals, and expert-curated pre-extracted features reveals that the cleaned dataset consistently outperformed expert-designed approaches by 8.40% in in-session validation and 12.4% in cross-session validation, challenging assumptions about conventional feature extraction methodologies. The identification of VEPC 10Hz as the optimal stimulus paradigm for maximum accuracy and Rest Closed Eyes as optimal for temporal stability establishes clear guidelines for different biometric application requirements.

XGBoost emerged as the superior classifier across all dataset variants and validation scenarios, while the uniform MFCC feature extraction successfully isolated preprocessing effects from feature engineering variations. The lenient preprocessing approach addresses critical gaps in current methodologies by balancing artifact removal with preservation of subject-specific neural signatures essential for both immediate identification accuracy and long-term temporal stability.

Cross-session validation highlights substantial temporal challenges for EEG-based biometrics, with performance dropping from 97% in-session to 34% cross-session. Despite this degradation, lenient preprocessing remains consistently beneficial across validation settings, supporting its practicality for deployment. Notably, the Rest Closed Eyes condition achieves 52.00% cross-session accuracy, suggesting a useful operating paradigm when temporal stability is prioritized over maximum in-session accuracy.

Beyond empirical performance, the impact of this work lies in showing that EEG preprocessing should be designed not only to suppress artifacts but also to preserve discriminative neural signatures. This shift in perspective establishes a foundation for developing deployable EEG biometric systems that are accurate, robust, and computationally feasible. The framework presented here has implications for secure authentication, neurotechnology, and personalized medicine, where non-invasive and reliable subject identification methods are increasingly needed.

### Future recommendations

Based on our findings, we identify three major research themes that are critical for advancing EEG-based biometric systems:

#### Temporal stability and longitudinal validation

The most pressing research need involves addressing the temporal stability challenges revealed by our cross-session analysis. Longitudinal validation studies spanning weeks to months are essential to evaluate the persistence of biometric signatures under different preprocessing conditions. Research should focus on developing adaptive preprocessing strategies that can accommodate temporal changes in neural patterns while maintaining biometric accuracy.

Investigation of optimal recording intervals and session scheduling protocols will inform practical deployment strategies. Additionally, research into personalized preprocessing approaches that adapt to individual temporal stability patterns could significantly improve cross-session performance. Critically, future studies should ensure data quality control measures to prevent excessive artifact contamination, as observed in many BED dataset sessions, which significantly contributes to reduced cross-session accuracy and limits the effectiveness of any preprocessing approach.

Advanced pre-processing techniques represent another critical area, including machine learning-guided preprocessing optimization that learns optimal parameters for individual subjects^24^ over time, hybrid approaches combining multiple artifact removal strategies based on temporal stability assessment, and real-time quality monitoring systems that can detect and compensate for temporal changes in signal characteristics while maintaining stringent data quality standards.

#### Real-world implementation and optimization

The transition from laboratory validation to practical deployment requires focused research on real-time implementation challenges. This includes developing efficient algorithms for real-time artifact detection and removal, investigating minimal preprocessing approaches that maintain biometric accuracy while reducing computational complexity, and optimizing battery life and power consumption for mobile EEG systems.

Security and privacy considerations become paramount for practical deployment, requiring investigation of template protection strategies for neural biometric data, development of privacy-preserving preprocessing and classification approaches, assessment of vulnerability to presentation attacks, and evaluation of secure transmission protocols for neural signals.

Stimulus paradigm optimization offers significant potential, building on our finding that Rest Closed Eyes provides optimal cross-session stability. Research should develop adaptive stimulus selection that chooses optimal paradigms based on individual temporal stability profiles, investigate hybrid approaches combining multiple stimulus types, and ensure user comfort and acceptance in practical applications.

#### Theoretical framework development

Fundamental research should focus on developing comprehensive theoretical models of how preprocessing decisions affect both immediate classification performance and long-term temporal stability. This includes mathematical frameworks for quantifying information loss during preprocessing, development of signal quality metrics specifically optimized for biometric applications, and investigation of optimal feature spaces for both in-session and cross-session identification.

Multi-modal integration represents an important theoretical and practical direction, combining EEG with other physiological signals to enhance both accuracy and temporal stability. Research should investigate how preprocessing strategies affect the integration of multiple biometric modalities and whether different preprocessing approaches are optimal for different temporal scales.

### Implications for practice

The findings from this study establish clear practical guidelines for EEG-based biometric system development. The lenient preprocessing approach provides an optimal balance between artifact removal and information preservation for both in-session and cross-session applications. For maximum accuracy applications, VEPC 10Hz stimulation offers the most effective paradigm for subject identification, while for temporal stability applications, Rest Closed Eyes emerges as the optimal choice.

XGBoost emerges as the recommended classifier across all validation scenarios, and the demonstrated effectiveness using consumer-grade hardware validates the practical viability of this approach for widespread deployment. The consistent preprocessing benefits observed across both in-session and cross-session validation provide confidence in the robustness of these recommendations.

These results challenge the conventional wisdom that aggressive preprocessing always improves EEG analysis, instead advocating for nuanced approaches that preserve the neural signatures essential for both immediate biometric identification and long-term temporal stability. The comprehensive framework established by this research provides a foundation for developing robust, accurate, and temporally stable EEG-based biometric systems suitable for real-world security and authentication applications.

## Data Availability

The BED dataset analyzed in this work is hosted on Zenodo (https://doi.org/10.5281/zenodo.4309472). Access to the dataset is subject to approval by the dataset maintainers; researchers may request access via the Zenodo record page. Additional processed data and analysis outputs generated during this study are available from the corresponding author upon reasonable request.
